# Implicit Detection of Poetic Harmony by the Naïve Brain

**DOI:** 10.3389/fpsyg.2016.01859

**Published:** 2016-11-25

**Authors:** Awel Vaughan-Evans, Robat Trefor, Llion Jones, Peredur Lynch, Manon W. Jones, Guillaume Thierry

**Affiliations:** ^1^School of Psychology, Bangor UniversityBangor, UK; ^2^School of Welsh, Bangor UniversityBangor, UK; ^3^Canolfan Bedwyr, Bangor UniversityBangor, UK

**Keywords:** language, neuroaesthetics, poetry, event-related potentials, P3b

## Abstract

The power of poetry is universally acknowledged, but it is debatable whether its appreciation is reserved for experts. Here, we show that readers with no particular knowledge of a traditional form of Welsh poetry unconsciously distinguish phrases conforming to its complex poetic construction rules from those that violate them. We studied the brain response of native speakers of Welsh as they read meaningful sentences ending in a word that either complied with strict poetic construction rules, violated rules of consonantal repetition, violated stress pattern, or violated both these constraints. Upon reading the last word of each sentence, participants indicated sentence acceptability. As expected, our inexperienced participants did not explicitly distinguish between sentences that conformed to the poetic rules from those that violated them. However, in the case of orthodox sentences, the critical word elicited a distinctive brain response characteristic of target detection –the P3b– as compared to the other conditions, showing that speakers of Welsh with no expertise of this particular form of poetry implicitly detect poetic harmony. These results show for the first time that before we even consider literal meaning, the musical properties of poetry speak to the human mind in ways that escape consciousness.

## Introduction

T. S. Eliot famously argued that “genuine poetry can communicate before it is understood” (Scofield, 1988; p. 2). Was this an attempt to provoke controversy or can some aspects of poetry indeed be processed implicitly and independently of meaning? Poetry is a literary expression of feelings, thoughts, and ideas, traditionally accentuated by metric constraints, rhyme, and alliteration. Recent scientific research looking into the effects of poetry has highlighted emotional responses to rhyme (Obermeier et al., 2013) and better memory recall as a result of alliteration (Hanauer, 2001; Lea et al., 2008). Rhyme violations, in particular, have been shown to increase pupillary responses (Scheepers et al., 2013) and modulate the amplitude of the N400, a brain potential index of semantic processing (Hoorn, 1996). Whilst there is little doubt that some poetic forms, often centuries old, impact human cognition (see Jacobs, 2015, for a recent review), we have yet to discover the extent to which such sensitivity may rely on automatic and implicit neural processing.

Here, we investigated event-related brain potentials (ERPs) elicited by the final word of sentences written in *Cynghanedd* (‘harmony’ in Welsh), an ancient poetic form that requires precise consonantal repetition (and/or internal rhyme) in conjunction with distinct stress patterns (Greene, 2012). In certain sub-types of Cynghanedd, consonants are repeated across the first and second parts of the line, and are always in the same order: *A* ***d****ae****th*** *i* ***b****en* | ***d****ei****th****io* ***b****yd* (‘And it came to an end | traveling the world,’ as cited in Llwyd, 2010, critical consonants in bold). A line such as ^∗^*A* ***d****ae****th*** *i* ***b****en* | ***d****ei****th****io****c****wm* (‘And it came to an end | traveling the valley’) features a ‘c’ rather than a ‘b,’ which constitutes a consonantal repetition violation. Traditional Cynghanedd rules also dictate a precise stress pattern: *Ei****n*** ***ll****u****n****iaeth* | *a’****n*** ***ll****awe****n****ydd* (‘Our sustenance and joy,’ Llwyd, 2010, stress vowels underlined and critical consonants in bold). In contrast, the line ^∗^*Ei****n*** ***ll****u****n****iaeth | a’****n*** ***ll****u* ***n****ewydd* (‘Our sustenance and new host’) violates traditional rules because ‘*n’* in part one comes after the stress, but ‘*n*’ in part two precedes the final stress. Cynghanedd sentences thus consist of foregrounding features at the sublexical (phonological salience) and lexical (stress pattern) levels (Jacobs, 2015). Each of these features is known to independently influence aesthetic appreciation (e.g., Aryani et al., 2013; Chen et al., 2016), but their interactive effect is unclear. In the present investigation, test sentences were constructed which either adhered to the rules of Cynghanedd, or violated its rules in terms of consonantal repetition, stress pattern, or both consonantal repetition and stress pattern (**Table 1**). Each condition was pseudo-randomly presented in equal proportion, resulting in an oddball paradigm with Cynghanedd-orthodox sentences occurring only 25% of the time.

**Table 1:** Experimental conditions.

Sentence	Rule adherence	Condition label
Y geiriau brwd ger y bryn	Consonantal repetition+Stress pattern+	Cynghanedd
Y geiriau brwd ger y bont	Consonantal repetition-Stress pattern+	Consonantal violation
Y geiriau brwd ger y border	Consonantal repetition+Stress pattern-	Stress violation
Y geiriau brwd ger y clawdd	Consonantal repetition-Stress pattern-	Double violation


*English translation: The fervent words near the hill/trees/border/bank.*

The P3b is an ERP component commonly observed during oddball paradigms thought to reflect a context-updating process whereby a comparison is made between the currently processed stimulus, and the previous representation held in working memory (see Polich, 2007, for a review). We anticipated that participants would show greater P3b amplitudes when singling out the infrequent target combination of consonantal repetition and stress pattern conforming to Cynghanedd from the other three non-Cynghanedd conditions. We were keen to know, however, whether such potential detection of the Cynghanedd-orthodox targets would be accompanied by signs of conscious evaluation as indexed by behavioral data and at debriefing.

## Materials and Methods

### Participants

Twenty-five fluent native speakers of Welsh (9 males; 16 females), with no prior knowledge of the rules of Cynghanedd, were included in the analysis. Of the initial participant pool, one participant was excluded due to prior knowledge of Cynghanedd and its underlying rules; two participants were excluded as they had too few uncontaminated epochs per condition; and a further four participants were removed as a result of overall excessive noise in the data. All participants possessed normal or corrected-to-normal vision. Ethical approval was granted by the School of Psychology, Bangor University ethics committee, and participants gave written consent before the experiment session started.

### Stimuli and Procedure

Experimental sentences belonged to 36 sets each consisting of four sentences, resulting in a total of 144 sentences. Twenty-five percent of the experimental sentences followed the rules of Cynghanedd whilst the remaining 75% violated the Cynghanedd rules in terms of consonantal repetition (25%), stress pattern (25%), or both consonantal repetition and stress pattern (25%; see **Table 1**). The experiment thus conformed to a classical oddball paradigm with Cynghanedd as the target condition. Where possible, sentence final words were rotated across conditions. However, due to the strict rules of Cynghanedd, it was not possible to fully rotate all items between conditions. Word frequency (from the *Cronfa Electroneg o Gymraeg*; Ellis et al., 2001) and length did not differ significantly between conditions [*F*(3,140) = 1.86, *p* = 0.14; *F*(3,140) = 0.76, *p* = 0.52].

**FIGURE 1:**
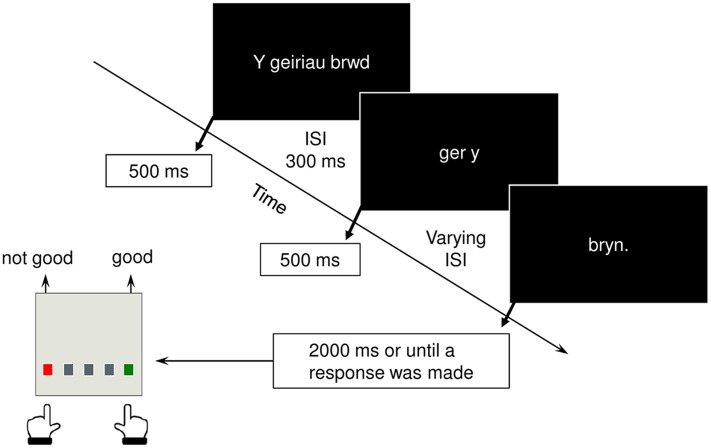
**Structure of an experiment trial and response required from participants**.

Participants viewed all 144 sentences in three sections, segmented such that they adhered to the natural rhythm of the Cynghanedd line, with the final, critical word presented in isolation. On each trial, the first two segments were presented for 500 ms each, with an inter-stimulus interval (ISI) of 300 ms. A varying ISI (ranging between 400 and 700 ms) was used between the second segment and the sentence final word, which remained on the screen for a maximum of 2000 ms, or until a response was made, whichever was the shortest (**Figure 1**). Presentation order was pseudorandomized, such that sentences from the same sentence set never appeared in the same experimental block. Upon presentation of the final word, participants were asked to indicate as quickly and as accurately as possible, whether or not the sentence sounded ‘good’ by pressing designated buttons on a serial response box. Upon completion of the experimental task, participants were presented with a list of the 36 sentence sets and were asked to rank the sentences in each set in a decreasing order of preference (1 = most preferred; 4 = least preferred).

### ERP Recording

Electrophysiological data was recorded from 32 Ag/AgCl electrodes set according to the extended 10–20 convention at a rate of 1 kHz in reference to the left mastoid. The electroencephalogram (EEG) activity was filtered online with a band-pass filter between 0.1 and 200 Hz and again offline with a band-pass zero-phase shift filter set between 0.1 and 20 Hz. Eye blink artifacts were modeled and mathematically corrected (Gratton et al., 1983) and remaining artifacts were removed manually. Epochs ranging from -100 to 1,000 ms after the onset of the target word were extracted from continuous EEG recordings. Epochs with activity exceeding ±75 μV at any electrode site were automatically discarded. There was a minimum of 30 epochs per condition for every participant. Baseline correction was performed in reference to pre-stimulus activity, and individual averages were digitally re-referenced to the algebraic mean of the left and right mastoids.

### Data Analysis

For the online categorization task, the percentage of ‘good’ responses was analyzed by means of a one-way repeated measures analysis of variance (ANOVA) with ‘Sentence Type’ (Cynghanedd, Consonantal violation, Stress violation, Double violation) as an independent variable. Reaction times were analyzed by means of a 2 (Categorization: ‘good,’ ‘not good’) × 4 (Sentence Type: Cynghanedd, Consonantal violation, Stress violation, Double violation) repeated measures ANOVA.

For the offline ranking task, responses were scored such that they were given a 1 if they correctly ranked Cynghanedd sentences as the ‘best’ sentence, and a 0 if they did not. Responses were then analyzed by means of a one-sample *t*-test.

For the ERP data, P3b mean amplitude was predictively extracted between 240 and 340 ms at six electrodes where the P3b is known to be maximal in amplitude (CP3, CPz, CP4, P3, Pz, P4) and maximal sensitivity was verified by inspecting the global field power produced across the scalp in the Cynghanedd condition. P3b mean amplitudes were analyzed by means of a one-way repeated measures ANOVA with ‘Sentence Type’ (Cynghanedd, Consonantal violation, Stress violation, Double violation) as an independent variable. *Post hoc* tests were conducted using Bonferroni corrections.

## Results

### Behavioral Results

#### Online Categorization Task

**FIGURE 2:**
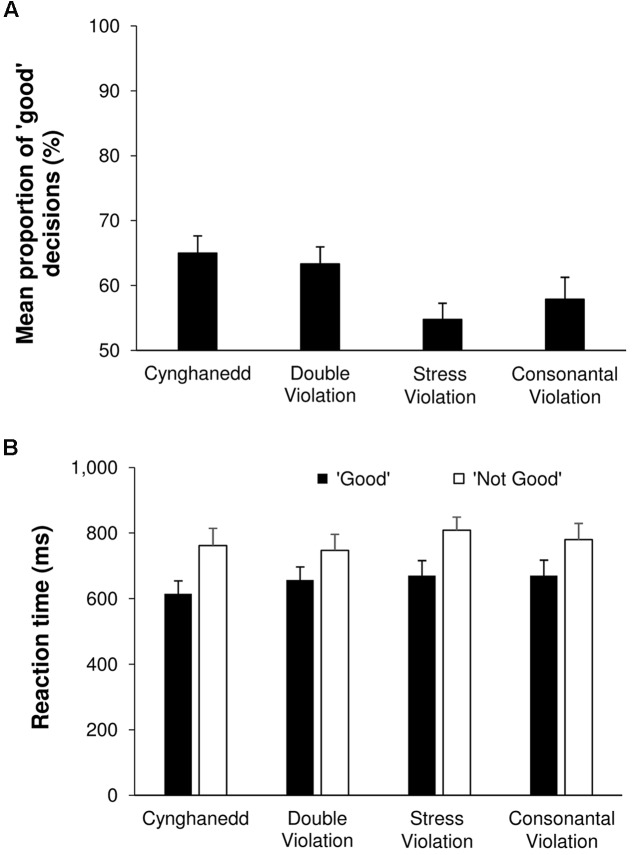
**Online categorization results.** **(A)** Sentence categorization performance. **(B)** Reaction times.

We found a significant main effect of Sentence Type; *F*(3,72) = 8.63, *p* < 0.001, *$\eta_{p}^{2}$* = 0.26 (**Figure 2A**.). Pairwise comparisons revealed that Cynghanedd sentences were more likely to be categorized as ‘good’ (*M* = 65%; 95% CI [60, 70]) compared with Consonantal violation sentences (*M* = 58%; 95% CI [51, 65]; *p* = 0.005) and Stress violation sentences (*M* = 55%; 95% CI [50, 60], *p* < 0.001), but not Double violation sentences (*M* = 63%; 95% CI [58, 69], *p* = 0.38). Furthermore, Double violation sentences were more likely to be categorized as ‘good’ than Consonantal violation sentences (*p* = 0.04) and Stress violation sentences (*p* = 0.001). Comparisons of categorization score against chance revealed that responses significantly differed from chance for Cynghanedd, the Double violation condition, and the Consonantal violation condition [*t*(24) = 2.325, *p* = 0.029], but not the Stress violation condition [*t*(24) = 1.905, *p* = 0.069]. Critically, whereas the greater than chance performance in the Cynghanedd condition was felicitous (these were the Cynghanedd-orthodox sentences), it was infelicitous in the Double violation and the Consonantal violation conditions.

For the reaction time data, a main effect of Categorization was found [*F*(1,24) = 33.58, *p* < 0.001, *$\eta_{p}^{2}$* = 0.58; **Figure 2B**]: Sentences that were perceived as ‘good’ were responded to faster (*M* = 653 ms, 95% CI [568, 738]) than sentences perceived as ‘not good’ (*M* = 774 ms, 95% CI [678, 869]). There was also a main effect of Sentence Type [*F*(3,72) = 3.24, *p* = 0.03, *$\eta_{p}^{2}$* = 0.12], but none of the corrected pairwise comparisons reached significance.

#### Offline Sentence Ranking Task

A one sample *t*-test revealed that participants did not rank Cynghanedd sentences as the best option significantly better than chance [*M*_accuracy_ = 28%; *t*(24) = 1.87, *p* = 0.07]. Since this result was approaching significance, we further tested whether participants showed any inclination to rank Cynghanedd sentences in the top two choices by coding the response as 1 if Cynghanedd sentences were ranked 1st or 2nd, or as 0 if Cynghanedd sentences were ranked 3rd or 4th. In this case, a one sample *t*-test revealed that participants did perform significantly greater than chance on this task [*M*_accuracy_ = 62%; *t*(24) = 6.93, *p <* 0.001].

#### Electrophysiological Data

**FIGURE 3:**
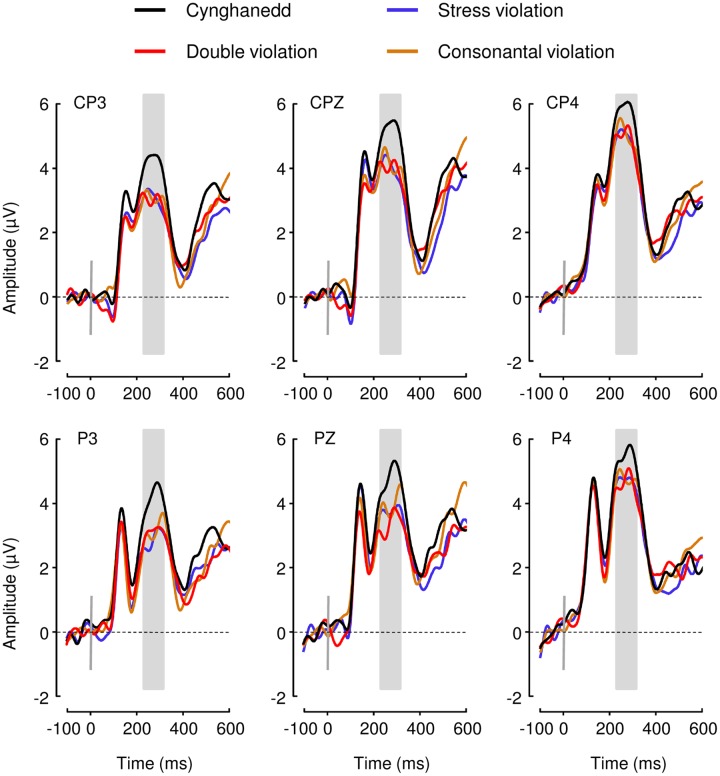
**Event-Related Brain Potential (ERP) results.** P3b mean amplitudes elicited by all four sentence types were computed and compared between 240 and 340 ms after the onset of the final word (gray box).

**FIGURE 4:**
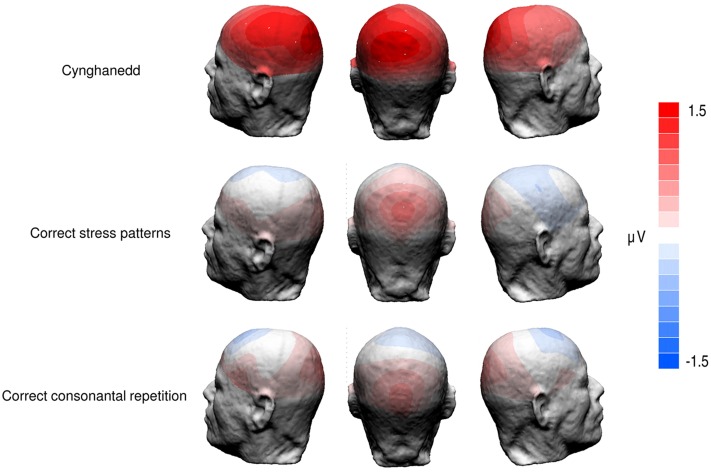
**Topographic maps of ERP difference waves in the P3b analysis window (240–340 ms after the onset of the final word).** *Cynghanedd* topographies depict differences between Double violation and Cynghanedd conditions. *Correct stress patterns* topographies depict differences between Double violation and Consonantal violation conditions. *Correct consonantal repetition* topographies depict differences between Double violation and Stress violation conditions.

We found a significant main effect of Sentence Type; *F*(3,72) = 3.149, *p* = 0.03, *$\eta_{p}^{2}$* = 0.12; with Cynghanedd sentences eliciting greater mean amplitudes (*M* = 5.93, 95% CI [4.86, 7.01) than Consonantal violation sentences (*M* = 5.01, 95% CI [3.92, 6.10]; *p* = 0.01), Stress violation sentences (*M* = 4.88, 95% CI [3.58, 6.17]; *p* = 0.002), and Double violation sentences (*M* = 5.00, 95% CI [3.90, 6.09]; *p* = 0.007), respectively (**Figure 3**). Analyses in earlier time windows (P1 and N1) did not show any significant differences as a result of the experimental conditions. As expected the distribution of the effect was centroparietal (**Figure 4**). Furthermore, the topographic maps show that participants were not sensitive to the consonantal repetition and stress pattern rules when presented independently; rather, they were only sensitive to constructions that complied with *both* consonantal repetition and stress pattern rules.

Upon visual inspection, the topography of the P3 appeared to be right-lateralized, whilst the experimental effect seemed more left-lateralized. In order to determine whether the interaction was significant, we conducted an additional ANOVA, with Sentence Type (Cynghanedd, Consonantal violation, Stress violation, Double violation) and ‘Laterality’ (Left [CP3;P3], Right [CP4;P4]) as independent variables. We found a significant effect of Laterality; *F*(1,24) = 27.66, *p* < 0.001, *$\eta_{p}^{2}$* = 00.54, with greater P3b mean amplitudes elicited on the Right (*M* = 6.05, 95% CI [5.01, 7.09]) than on the Left (*M* = 4.37, 95%CI [3.39, 5.36]). The Sentence Type ^∗^ Laterality interaction did not reach significance [*F*(1,24) = 1.05, *p* = 0.377, *$\eta_{p}^{2}$* = 0.04], however, indicating that the experimental effect was not modulated by electrode site.

## Discussion

Here, we investigated whether naïve readers of a traditional form of Welsh poetry are able to unconsciously distinguish phrases conforming to its poetic construction rules from those that violate them. In line with our predictions, words correctly completing a sentence in Cynghanedd elicited significantly greater P3b mean amplitudes than words completing other sentence types, indicating a shift of attention associated with target recognition (Polich, 2007).

The P3b modulation observed here had a typical centroparietal distribution and a time-range comparable to that observed in simple target detection tasks, consistent with the classic P3b effect (Knight, 1996). Thus, participants’ brains treated correct completion words as targets and implicitly categorized Cynghanedd-orthodox sentences as sounding ‘good’ compared to sentences violating its construction rules. Strikingly, however, and in contrast with ERP results, participants showed no overt knowledge or conscious awareness of Cynghanedd rules in the online categorization task since (a) they failed to discriminate between Cynghanedd and Double Violation sentences, and (b) their performance was either at chance level (Stress violation condition) or infelicitous with regard to Cynghanedd rules in the other violation conditions. There was some differentiation between sentence types, with participants rating Cynghanedd sentences as sounding better than those from single violation conditions. It is possible that this difference occurred due to participants perceiving the rule violations in these conditions, however, this interpretation cannot account for the fact that participants did not consider Cynghanedd sentences as sounding better than Double violation sentences. Participants did, however, demonstrate a preference toward Cynghanedd sentences during the offline judgment task. Given that the ranking task was of a very different nature to the online task (involving direct comparison between the different alternatives of each sentence) and that it was not time constrained, it is highly likely that participants changed cognitive strategy in this task, and focused on elements of the stimuli that were not attended to during the online categorization task.

Interestingly, the results of the online decision task are somewhat incongruent with recent research emphasizing the influence of foregrounded features on aesthetic appreciation (Aryani et al., 2013). For example, Aryani et al. (2013) demonstrated, via use of a text analysis tool, that the salience of particular sublexical features (e.g., phonological repetition) correlates with the semantic and aesthetic properties of poetic phrases. Given that a ‘sound good’ judgment could be influenced by such foregrounding properties, Cynghanedd and Stress violation sentences should be judged as ‘good’ more than the other two sentence types, but this was not the case in our data. Whilst participants considered Cynghanedd sentences as sounding better than those from single violation conditions, they did not consider Cynghanedd sentences as sounding better than Double violation sentences. This finding could be interpreted in one of two ways; (1) the consonantal repetition manipulation was too subtle to influence participants’ explicit judgments, or (2) the ‘sound good’ decision task implemented in this study did not depend on the affective qualities of the repeated phonemes. In addition, the ERP results suggest that appreciation of Cynghanedd depends on a combination of subtle consonantal repetition *and* stress pattern, rather than consonantal repetition alone.

The P3b effect observed here may be considered counter-intuitive, since P3b amplitude is classically reduced with repeated occurrences of stimuli. Here, the presence of consonantal repetition patterns in the Cynghanedd condition may have been expected to reduce the amplitude of the P3b rather than increase it. Thus, the enhanced P3b response to Cynghanedd appears to indicate a kind of attentional orienting response, specifically when both the stress pattern and consonantal repetition rules are observed, thus making this particular sentence a target. This is congruent with recent electrophysiological evidence showing that lyrical stanzas that contain consistent meter *and* rhyme facilitate processing compared with those that contain only one, or neither of these patterns (Obermeier et al., 2016). Another recent study has shown that electrophysiological responses to poetry can be modulated by prosodic elements (e.g., rhyme) alone (Chen et al., 2016). Our findings are somewhat incongruent with this conclusion, since stress pattern alone failed to generate a main effect on P3b mean amplitudes.

Recent eye-tracking studies have also shown that literary stylistic features in sentences increase attentional engagement (see Jacobs, 2015, for a review). Our data crucially show that this attentional orienting effect occurs as early as 240 ms, after stimulus onset and is therefore likely to reflect implicit processing. Recall that participants were unable to overtly identify the Cynghanedd forms, and we found no correlation between reaction times and P3b mean amplitudes, contra previous findings (Conroy and Polich, 2007; Ramchurn et al., 2014; but see McCarthy and Donchin, 1981). Thus, whereas previous studies have shown that the explicit, aesthetic appreciation of poetry can be linked to implicit responses (e.g., Jacobs, 2015; Obermeier et al., 2016), the current findings provide the first tangible evidence that this link is permeable: our participants were able to *implicitly* detect correct poetic forms, even though they could not explicitly differentiate between conditions (cf. Renault et al., 1989).

Furthermore, despite the relatively complex nature of the processes underlying the decision task, the observed P3b had a latency akin to that typical of simple shape-matching tasks (Kok, 2001), occurring much earlier than typical responses to linguistic stimuli (Kutas and Hillyard, 1980). This suggests that spontaneous recognition of poetic harmony is a fast, sublexical process, and is not strategic nor cognitively effortful. Finally, our findings show that the brain responds to *combinations* of poetic – or foregrounding – features at the sublexical (phonological salience) and the lexical (stress pattern) levels (cf. Jacobs, 2015 4 × 4 model of neurocognitive poetics). That is, our data suggest that the interactive effects of poetic features are more potent than that of features presented in isolation.

Taken together, our results demonstrate the ability of the human brain to process poetic forms spontaneously, quickly, and implicitly, in the absence of any formal knowledge or instruction regarding underlying construction rules. This study shows for the first time that before we even consider literal meaning, the musical properties of poetry instinctively speak to the human mind in ways that escape consciousness.

## Author Contributions

GT, RT, LJ, and PL designed research; AV-E performed research; AV-E and GT analyzed data; AV-E, MJ, and GT wrote the paper; RT, LJ and PL contributed to the paper.

## Conflict of Interest Statement

The authors declare that the research was conducted in the absence of any commercial or financial relationships that could be construed as a potential conflict of interest.
